# Developing a *Hazomalania voyronii* Essential Oil Nanoemulsion for the Eco-Friendly Management of *Tribolium confusum, Tribolium castaneum* and *Tenebrio molitor* Larvae and Adults on Stored Wheat

**DOI:** 10.3390/molecules26061812

**Published:** 2021-03-23

**Authors:** Nickolas G. Kavallieratos, Erifili P. Nika, Anna Skourti, Nikoletta Ntalli, Maria C. Boukouvala, Catherine T. Ntalaka, Filippo Maggi, Rianasoambolanoro Rakotosaona, Marco Cespi, Diego Romano Perinelli, Angelo Canale, Giulia Bonacucina, Giovanni Benelli

**Affiliations:** 1 Laboratory of Agricultural Zoology and Entomology, Department of Crop Science, Agricultural University of Athens, 75 Iera Odos Str., 11855 Athens, Attica, Greece; erifilinika@aua.gr (E.P.N.); annaskourti@aua.gr (A.S.); mbouk@aua.gr (M.C.B.); p1171915@aua.gr (C.T.N.); 2 Laboratory of Efficacy Assessment of Pesticides, Scientific Directorate of Pesticides’ Assessment and Phytopharmacy, Benaki Phytopathological Institute, 8 Stefanou Delta Str., 14561 Kifissia, Attica, Greece; nntali@agro.auth.gr; 3 School of Pharmacy, University of Camerino, 62032 Camerino, Italy; filippo.maggi@unicam.it (F.M.); marco.cespi@unicam.it (M.C.); diego.perinelli@unicam.it (D.R.P.); giulia.bonacucina@unicam.it (G.B.); 4 Centre National d’Application de Recherches Pharmaceutiques, Ambodivoanjo Ambohijatovo, Rue RP Rahajarizafy Analamahitsy, BP 702, 101 Antananarivo, Madagascar; rravalison@gmail.com; 5 Ecole Supérieure Polytechnique d’Antananarivo, University of Antananarivo, BP 1500, 101 Antananarivo, Madagascar; 6 Department of Agriculture, Food and Environment, University of Pisa, Via del Borghetto 80, 56124 Pisa, Italy; angelo.canale@unipi.it (A.C.); giovanni.benelli@unipi.it (G.B.)

**Keywords:** botanical-based insecticide, cereals, green grain protectant, essential oil nanoformulation, stored-product beetles, Tenebrionidae

## Abstract

Most insecticides commonly used in storage facilities are synthetic, an issue that generates concerns about food safety and public health. Therefore, the development of eco-friendly pest management tools is urgently needed. In the present study, a 6% (*w/w*) *Hazomalania voyronii* essential oil-based nanoemulsion (HvNE) was developed and evaluated for managing *Tribolium confusum*, *T. castaneum*, and *Tenebrio molitor*, as an eco-friendly wheat protectant. Larval and adult mortality was evaluated after 4, 8, and 16 h, and 1, 2, 3, 4, 5, 6, and 7 days, testing two HvNE concentrations (500 ppm and 1000 ppm). *T. confusum* and *T. castaneum* adults and *T. molitor* larvae were tolerant to both concentrations of the HvNE, reaching 13.0%, 18.7%, and 10.3% mortality, respectively, at 1000 ppm after 7 days of exposure. However, testing HvNE at 1000 ppm, the mortality of *T. confusum* and *T. castaneum* larvae and *T. molitor* adults 7 days post-exposure reached 92.1%, 97.4%, and 100.0%, respectively. Overall, the HvNE can be considered as an effective adulticide or larvicide, depending on the target species. Our results highlight the potential of *H*. *voyronii* essential oil for developing green nanoinsecticides to be used in real-world conditions against key stored-product pests.

## 1. Introduction

The confused flour beetle, *Tribolium confusum* (Jacquelin du Val) (Coleoptera: Tenebrionidae), is a cosmopolitan secondary stored-product pest of high economic importance [1]. It has been reported to infest 119 different commodities [2]. The contamination of infested stored products by body fragments and toxins (e.g., methyl-1,4-benzoquinone, ethyl-1,4-benzoquinone, methoxybenzoquinone) may have a negative impact on consumers [3,4]. Due to its ability to infest processed commodities, it is usually found in mills, bakeries, pet shops, and storage units [2]. Recently, Kavallieratos et al. [5] reported that the larval and pupal development period is a complex phenomenon depending on the geographical origin of *T. confusum* and the type of infested commodity.

The red flour beetle, *Tribolium castaneum* (Herbst) (Coleoptera: Tenebrionidae), is a key secondary pest of stored products that is globally distributed [1]. It has been reported to infest 246 different commodities, an issue that makes it one of the most polyphagous stored-product insect pests [2]. It can be routinely found in mills, storage units, pet stores, and retail stores [2]. As in the case of *T. confusum*, *T. castaneum* produces quinones that cause skin irritation [6]. Skourti et al. [7,8] recently outlined that different levels of temperature and different types of commodities, chiefly alter the immature developmental period.

The yellow mealworm beetle, *Tenebrio molitor* L. (Coleoptera: Tenebrionidae), is one of the biggest stored-product insects. It is categorized as a secondary pest and a scavenger [1]. It infests fewer commodities (i.e., at least 46) compared to the other two species [2]. *Tenebrio molitor* can be easily found in flour mills, storage units, and food shops [2]. Apart from being a pest of stored products, it is reared as pet food for birds, fish, and reptiles [1,9,10], and–more recently–widely considered as a promising food for humans, while it degrades polystyrene and plastic waste [11]. *Tenebrio molitor* contaminates the commodities with quinones, but it is not as severe as the contamination by the species belonging to the *Tribolium* genus [10]. Anyway, it can cause allergic reactions in humans [6]. It can complete the biological cycle in only 30 days, while as an adult, it can survive up to two years [1,3].

It is well known that tenebrionids can develop resistance to several insecticides [12,13,14]. Therefore, new insecticidal formulations are necessary to be developed [15,16,17,18]. Among natural products with efficacy against stored-product insects, essential oils (EOs) and their main constituents have been revealed to be promising [18,19,20]. However, in real-world conditions, EOs need to be encapsulated in micro- and nanoformulations to enhance their persistence and physio-chemical stability while maintaining their biological properties [21,22]. Nanoemulsions (NEs) can be considered one of the most promising ways for the encapsulation and formulation of EOs. Nanoemulsions are kinetically stable oil droplets in water systems with a surfactant-to-oil ratio (SOR) ranging from 1 to 2 [23], and a droplet diameter <100 nm. Thanks to the reduced size of the droplets of the internal phase and the consequent increment of the surface area, NEs allow a better interaction of the encapsulated compounds into the target site [23] by overcoming the EO’s poor physicochemical stability and solubility issues [24]. This strategy can boost the applicability of EOs as natural insecticides [25,26,27,28,29].

Therefore, EO-based NEs are alternative solutions for pest management for a wide spectrum of insects of public health importance, and crop pests as well. For example, Ghosh et al. [30] treated 3rd instar larvae of *Aedes aegypti* L. (Diptera: Culicidae) with different NE concentrations from *Ocimum basilicum* L. (Lamiaceae) EO. Larval mortality was completely suppressed even after 15 min of exposure at 200 mL of 10-fold diluted NE. Duarte et al. [31] tested the larvicidal efficacy of 5% (*w*/*w*) NE of *Rosmarinus officinalis* L. (Lamiaceae) EO against the 4th instar larvae of *Ae. aegypti*. Mortality levels reached 80% after 24 h and 90% after 48 h at 250 ppm. Recently, Benelli et al. [27] proposed a 6% (*w*/*w*) NE of *Carlina acaulis* L. (Compositae) root EO against the European grapevine moth, *Lobesia botrana* (Denis and Schiffermüller) (Lepidoptera: Tortricidae), reaching 50% and 90% mortality of 1st instar larvae with 9.04 and 17.70 µL/mL, respectively. In a further recent study, Pavela et al. [32] evaluated NEs based on *C. acaulis* EO and found that less than 1200 μL/L caused 90% mortality to 3rd instar larvae of *Culex quinquefasciatus* Say (Diptera: Culicidae).

However, there is little published research on the utilization of NEs based on EOs as grain protectants [16,33]. For instance, Hashem et al. [19] used an NE of *Pimpinella anisum* L. (Apiaceae) EO for the management *T. castaneum* adults, over a wide spectrum of NE concentrations, on cracked wheat kernels. Nevertheless, there are no data on the efficacy of *Hazomalania voyronii* (Jum.) Capuron (Hernandiaceae) EO-based NE against *T. confusum, T. castaneum*, and *T. molitor.* However, the bioactivity of the pure EO of *H. voyronii* has been recently studied [20], showing that the raw *H*. *voyronii* EO exerted a rather limited toxicity as a grain protectant against important stored-product beetles. Even on selected species and instars (e.g., *Trogoderma granarium* Everts (Coleoptera: Dermestidae) adults), mortality rates reached about 79% after 7 days of exposure at 1000 ppm. In this framework, one may hypothesize that a way to boost the efficacy of this EO may be to develop highly stable EO-based nanoformulations [23]. As a model EO prototype, here we used the one obtained from *H. voyronii*, a traditional Malagasy plant (e.g., it is used to heal wounds, the drinkable bark decoction of stems is used for the treatment of malaria) with documented insecticidal efficacy [34]. Perilla aldehyde, the major compound of the *H. voyronii* EO, is used as a flavouring component to baked foods, sweets, meat products, dressing for salads, sauces, salted vegetables, and beverages [35]. Furthermore, perilla aldehyde is a “generally recognized as safe” (GRAS) substance [36].

To validate the hypothesis formulated above, the objective of the present study was the development of a 6% (*w*/*w*) *H. voyronii* EO-based NE for the effective and eco-friendly management of larvae and adults of three major stored-product beetles (i.e., *T. confusum, T. castaneum*, and *T. molitor).* To assess the applied potential to protect stored grains, the effectiveness of this NE as a grain protectant was investigated in small environments mimicking real wheat storage conditions.

## 2. Results

### 2.1. Development and Characterization of H. voyronii EO-Based NE

**Figure 1:**
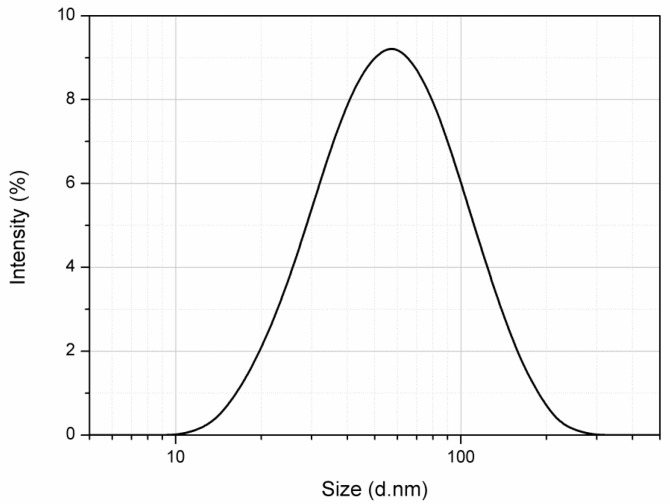
Size distribution (d.nm) of the prepared *Hazomalania voyronii*-based nanoemulsion (6% *w*/*w*) as obtained from dynamic light scattering.

After a preliminary screening, the quantitative composition of the *H*. *voyronii* NE was selected as follows: 6% (*w*/*w*) of the EO phase was emulsified in the aqueous medium containing 4% (*w*/*w*) of surfactant (Polysorbate 80). A high-energy method (i.e., high pressure homogenization) was employed at the pressure of 130 MPa to obtain *H. voyronii* EO-based NE characterized by oil droplets with a size in the nanometric range. From DLS analysis, in fact, the sample showed a monomodal size distribution with a mean diameter (Z-average) of 53.54 ± 0.20 nm and a polydispersity index of 0.340 ± 0.013 after preparation (Figure 1). The absence of oil droplets with a diameter above 1 µm confirms the formation of a true NE. Indeed, the sample appeared homogenous upon observation by optical microscope.

### 2.2. Insecticidal Efficacy

When the insecticidal efficacy of the 6% (*w*/*w*) *H*. *voyronii* NE was evaluated, between exposure intervals, all main effects were significant, while the associate interaction was not significant (Table 1). Within exposure intervals, the main effect as well as the interaction exposure x insect species-stage were significant, while the interactions exposure x NE concentration and exposure x NE concentration x insect species-stage were not significant (Table 1).

Concerning *T. castaneum* adults, the mortality caused by *H. voyronii* EO-based NE was 0.0% until the 1st day post-treatment, then reached 2.2% after 2 days of exposure for both tested NE concentrations (Table 2). The mortality remained low and did not exceed 12.5% and 18.7% at 500 ppm and 1000 ppm, respectively, after 7 days of exposure. Although the mortality of *T. castaneum* larvae did not exceed 10% at 500 ppm and 12.6% at 1000 ppm 16 h post-exposure, it reached 84.1% at 500 ppm and 97.4% at 1000 ppm, after 7 days of exposure.

Regarding *T. confusum* adults, the mortality remained at low levels (Table 3). After 4 days of exposure at 500 ppm, the mortality was 3.3%, and after 7 days it reached 10.3%. The mortality at 1000 ppm was not significantly higher than the one at 500 ppm, after 2 and 7 days of exposure reaching 3.3% and 13.0%, respectively. As far as *T. confusum* larval mortality is concerned, 5 days of exposure to the two NE concentrations led to significantly higher mortality at 1000 ppm than 500 ppm (i.e., 45.0% and 26.4%, respectively). The *H. voyronii* NE killed 59.3% of larvae at 500 ppm, and 92.1% of larvae at 1000 ppm, 7 days post-exposure.

Mortality of *T. molitor* adults was <90% after the 7th day of exposure (94.8%) at 500 ppm, while at 1000 ppm after the 6th day of exposure it was 93.5% (Table 4). Complete mortality of this life stage was achieved testing 1000 ppm of *H. voyronii* NE after 7 days post-exposure. No mortality of *T. molitor* larvae was noted testing 500 ppm and 1000 ppm, 2 days and 1 day post-exposure, respectively. At the end of the experimental period, the overall larval mortality did not exceed 5.8% at 500 ppm, and 10.3% at 1000 ppm.

## 3. Discussion

Our results indicate that the NE-based on *H. voyronii* EO (6% *w*/*w*) is effective against *T. castaneum, T. confusum*, and *T. molitor*. This nanosystem led to high mortality levels on different life stages of the tested species, even at 500 ppm. Concerning *T. molitor* adults, the mortality reached 100% at 1000 ppm, and 94.8% at 500 ppm, after 7 days of exposure. The mortality of larvae was very low, even at the highest tested concentration (1000 ppm), reaching 10.3% after 7 days of exposure. Earlier research has revealed that the adult stage is the most susceptible life stage of *T. molitor*. For example, Kavallieratos et al. [37] found that when 0.504 ppm deltamethrin, 5 ppm pirimiphos-methyl, 1000 ppm silicoSec (which is a diatomaceous earth (DE)), and 1 ppm spinosad were applied on stored wheat as grain protectants, high adult mortality rates were achieved (92.2%, 100%, 100%, and 94,4%, respectively), while they caused moderate larval mortality (10.0%, 71.1%, 43.3%, and 28.9%, respectively). Apart from the wheat, barley and maize have also been tested on the same formulations as grain protectants, and the results followed the same pattern, with larvae being more tolerant than adults. In addition, testing the efficacy of 5 ppm pirimiphos-methyl on barley in a wide range of temperature and relative humidity levels, a greater mortality of *T. molitor* adults was found, if compared to the larvae [38].

In an earlier study, the raw EO of *H. voyronii* showed relevant efficacy on *T. granarium* adults, but not on the larvae [20]. The raw EO caused 78.9% adult mortality at 1000 ppm, but only 15.6% at 500 ppm after 7 days of exposure. Larvae were more tolerant and the mortality rates at 500 ppm and 1000 ppm after 7 days of exposure did not exceed 4.4% and 15.6%, respectively. Although the raw *H. voyronii* EO does not allow an adequate control of *T. molitor* and *T. granarium* larvae, the suppression of the adult stage of both pests is a very important finding, as adults are the vehicle of reproduction [20].

In contrast, the results of the present study outlined that the adults of *T. castaneum* and *T. confusum* remained practically unaffected by being exposed to the *H. voyronii* EO-based NE, given that the highest adult mortality was 18.7% for *T. castaneum* at 1000 ppm and 13.0% at 1000 ppm for *T. confusum,* while the larvae had a mortality of 97.4% for *T. castaneum* at 1000 ppm and 92.1% at 1000 ppm for *T. confusum* after 7 days of exposure. Previous research on *T. castaneum* has reported that the adults are tolerant to several insecticides. Fumigation studies on the species of four EOs extracted from the plants *Lantana camara* L. (Verbenaceae), *Cymbopogon nardus* (L.) Rendle (Poaceae), *Cinnamomum zeylanicum* Blume (Lauraceae), and *Trachyspermum ammi* (L.) Sprague (Apiaceae) showed that for all the tested EOs and exposure times, *T. castaneum* adults needed relatively high EO volumes (e.g., 14.56, 37.52, 4.40, and 14.86 μL, respectively, for each EO after 72 h of exposure) than the larvae (e.g., 5.00, 4.13, 2.48, and 2.73 μL, respectively, for each EO after 72 h of exposure) [39]. When the d-strain of *T. castaneum* was treated with 1 mg/L and the r-strain with 2 mg/L phosphine (PH_3_), after 4 h of treatment, adults have been found more tolerant than the larvae. For both d- and r-strains of *T. castaneum,* after 24 h of exposure to <10 mg/L carbonyl sulphide (COS), the larvae were less tolerant than adults [40]. Earlier, Arthur [41] reported that larvae were less tolerant than the adults. Regarding contact toxicity, Deb and Kumar [42] reported that the *Artemisia annua* L. (Asteraceae) EO had greater larvicidal than adulticidal efficacy against *T*. *castaneum*. Mujeeb and Shakoori [43] suggested the larval stage as the preferable life stage to apply pirimiphos-methyl, outlining that this is the most susceptible stage. Adults of *T. confusum* were also more tolerant than the larvae. The natural insecticide silicoSec attained 100% larval mortality after 7 days of exposure, but when tested on adults it did not exceed 85% after 14 days of exposure [44]. Spinetoram and spinosad [45] had high larval mortality reaching 98.9% in a mixture of the two insecticides after 14 days on treated wheat kernels, while the adult mortality reached 67.8% for the same mixture after 14 days on treated wheat kernels. Similarly, studying the effectiveness of eight pyrrole derivatives, a better larvicide than adulticide action has been detected [46,47,48].

EO constituents have a wide spectrum of effectiveness on adults and larvae that could be partially explained by their different mode of actions [49]. For instance, perilla aldehyde, the main component of *H. voyronii* EO, showed insecticidal and inhibitory effects on the enzyme acetylcholinesterase (AChE) in *Drosophila suzukii* (Matsumura) (Diptera: Drosophilidae) [50], and it has been argued that its insecticidal activity could be related to the presence of exocyclic and endocyclic double bonds in the chemical structure [51]. Furthermore, 1,8-cineole and limonene, the other two main constituents of the *H*. *voyronii* EO, showed insecticidal efficacy against a wide spectrum of insects, including mosquitoes (e.g., *Culex pipiens* L. (Diptera: Culicidae), *Cx. quinquefasciatus*, *Ae. aegypti, Ae. albopictus* (Skuse) (Diptera: Culicidae)), houseflies (*Musca domestica* L. (Diptera: Muscidae)), and stored-product beetles (*Sitophilus granarius* (L.) (Coleoptera: Curculinonidae)) [52,53,54,55].

The significant toxicity of the EO-based NE developed here against *T. castaneum* and *T. confusum* larvae is important, since targeting the adults is crucial to achieve a major reduction of the overall population. Storage units can host several species in different developmental stages existing simultaneously [56,57,58,59]. Therefore, insecticides based on natural products such as EO-based NEs, which can manage a broad spectrum of stored-product insects, are highly desirable [16,33]. Our results showed that the *H. voyronii* EO-based NE is toxic against three tenebrionid species. Similarly, Hashem et al. [19] tested four different concentrations of a *P. anisum* EO-based NE against *T. castaneum* adults, showing that 7.5% and 10% *v*/*v* killed 51.2% and 74.3% of the exposed individuals, in comparison to the control after 9 days of exposure, respectively; 12 days post-exposure, the overall mortality rates reached 54.7% at 7.5% *v*/*v* and 81.3% at 10% v/v.

Our findings indicate that among the different species tested here, all belonging to the Tenebrionidae family, there is a wide variability of the performance of *H. voyronii* EO-based NE as a grain protectant. However, the level of efficacy depends on the life stage of the target species. A crucial factor for elevated effectiveness of insecticides is the timing of their application [7,60]. Therefore, the knowledge of the species and the life stage that infests grain commodities could maximize the management strategy if the *H. voyronii* EO-based NE is selected as a component of a management strategy against *T. castaneum, T. confusum*, and *T. molitor*.

## 4. Materials and Methods

### 4.1. Essential Oil

The dry bark of *H. voyronii* was obtained from trees growing in Kirindy Forest, Madagascar (coordinates: S 20°28′15.002″; E 44°17′56.06″; 62 m a.s.l.) in February, 2018. Then, it was subjected to hydrodistillation for 3 h using a Clevenger-type apparatus. The chemical composition was recently reported in our recent study by Benelli et al. [34]. The major components were oxygenated monoterpenes, namely perilla aldehyde (43%), 1,8-cineole (33.2%), and limonene (13%).

### 4.2. Insects

*Tribolium confusum* and *T. castaneum* were cultured on wheat flour and 5% brewer’s yeast, and *T. molitor* on a combination of oat bran and potato slices for additional moisture [61], at 30 °C, 65% relative humidity, and continuous darkness [37,62,63]. The founding colonies were acquired from storage facilities in Greece. The two *Tribolium* species were maintained in the Laboratory of Agricultural Zoology and Entomology, Agricultural University of Athens, since 2003, and *T. molitor* since 2014. For the tests, unsexed adults <2 weeks old, and larvae that were 3rd–4th instar old (for *T. confusum* and *T. castaneum*) and 10–14 mm long (for *T. molitor*) were used [37,62].

### 4.3. Commodity

Hard wheat, *Triticum durum* Desf. (var. Claudio) (Poales: Poaceae) clean and free of infestations and pesticides was used in the experiments. Prior to the bioassays, moisture content of the wheat kernels was 12.4%, calibrated by a moisture meter (mini GAC plus, Dickey-John Europe S.A.S., Colombes, France).

### 4.4. Development and Characterization of H. voyronii EO-Based Nanoemulsion

*Hazomalania voyronii* EO NE was obtained through a high-energy method, by using a high-pressure homogenizer according to the procedure reported by Cappellani et al. [64]. A 6% (*w*/*w*) of EO was added dropwise to 4% (*w*/*w*) Polysorbate 80 (Sigma-Aldrich) aqueous solution under high-speed stirring (Ultraturrax T25 basic, IKAfi Werke GmbH & Co.KG, Staufen, Germany) for 5 min at 9500 rpm. The obtained emulsion was then homogenized with a French Pressure Cell Press (American Instrument Company, Aminco, MY, USA) for four cycles at a pressure of 130 MPa. Visual inspection of the formulation was performed by using a polarizing optical microscope (MT9000, Meiji Techno Co. Ltd., Chikumazawa, Miyoshi machi, Iruma-gun Saitama, Japan) equipped with a 3-megapixel CMOS camera (Invenio 3S, DeltaPix, Smørum, Denmark) to assess NEs formation. Dynamic light scattering (DLS) analyses were then carried out to determine lipophilic internal phase droplets size using a Zetasizer Nano S (Malvern Instruments, Worcestershire, UK) equipped with backscattered light detector working at 173°. A sample of 1 mL was analysed at 25 °C, following a temperature equilibration time of 180 s. The analyses were performed in triplicate.

### 4.5. Insecticidal Assays

The NE based on *H. voyronii* EO (6% *w*/*w*) was tested at the concentrations of 500 μL/kg wheat (=500 ppm) and 1000 μL/kg wheat (=1000 ppm). The test concentrations were selected based on preliminary tests on the three tenebrionid species. Test solutions were prepared in water at the final volume of 750 μL, and sprays were performed on plates, where 0.25 kg wheat lots were laid out [20]. Additional lots of 0.25 kg wheat treated with (i) water and (ii) carrier control (4% *w*/*w* surfactant dispersed in water) served as controls. The spraying of the wheat lots was conducted by an AG-4 airbrush (Mecafer S.A., Valence, France) on different trays. Controls were sprayed using different AG-4 airbrushes. Then treated lots were transferred to 1-L glass containers and were shaken for 10 min to equally distribute the NE on the total quantity of wheat. The same procedure was followed for controls. From each treated lot or controls, three samples of 10 g each were obtained with different scoops. A Precisa XB3200D compact balance (Alpha Analytical Instruments, Gerakas, Greece) was used to weigh the samples on a filter paper that was unique for each sample. Then, samples were placed in glass vials (7.5 cm × 12.5 cm diameter and height). The caps of the vials had a 1.5 cm diameter circular opening in the middle, covered with gauze, to sufficiently aerate the content of the vials. The upper inner necks of the vials were covered by polytetrafluoroethylene (60 wt % dispersion in water) (Sigma-Aldrich Chemie GmbH, Taufkirchen, Germany), to assure that the beetles would remain in the vials. Thereafter, 10 larvae or adults of each tenebrionid species were separately transferred into the vials. The containers remained in incubators set to 30 °C and 65% RH for the whole experimental period. Mortality rates’ evaluation was accomplished by an Olympus stereomicroscope (SZX9, Bacacos S.A., Athens, Greece) at 57x total magnification after 4, 8, and 16 h and 1, 2, 3, 4, 5, 6, and 7 days of exposure, by gently nudging each individual insect using a fine brush (Cotman 111 No 000, Winsor and Newton, London, UK) to detect any movement. For each concentration of the NE and control, different brushes were used. The above procedure was repeated three times with new insects, wheat, and vials.

### 4.6. Data Analysis

Control mortality on wheat treated with water was low (<5%) for all insect species and life stages tested, therefore no correction was necessary. In contrast, the control mortality on wheat treated with the carrier control 4% *w*/*w*surfactant dispersed in water, was >5% for all tested species and life stages, ranging up to 17.8%. Therefore, mortality values were corrected by the Abbott formula (i.e., (1 − insect population in treated unit after treatment/insect population in control unit after treatment) × 100) [65]. Before conducting analysis, the mortality data were log(*x* + 1) transformed to normalize variance [66,67]. Statistical analyses were conducted by following the repeated-measures model [68]. Exposure interval was the repeated factor, and mortality was the response variable. The main effects were the concentration and insect species/developmental stage. The associated interactions of the main effects were considered in the analysis. All analyses were conducted using JMP v.14 software [69]. Means were separated using the Tukey–Kramer (HSD) test at 0.05 of significance [70]. The two-tailed *t*-test at *n*-2 *df* and 0.05 significance [71] was used to compare the two tested concentrations of *H. voyronii* EO-based NE at each species or life stage.

## 5. Conclusions

To eco-friendly manage several important insect pests of stored products, a combination of the *H. voyronii* EO-based NE with other natural insecticides (e.g., DEs) could potentially provide an enhanced level of protection for stored durable commodities against multi species infections. For example, the use of DE or natural zeolite as grain protectants caused 100% mortality on *T. castaneum* adults after 14 or 21 days of exposure, respectively [72]. A combination of *H. voyronii* EO-based NE with DE or natural zeolite could enhance the likelihood of the management of *T. castaneum, T. confusum,* and *T. molitor,* regardless of their being adults or larvae, an issue that merits further investigation. Furthermore, Athanassiou et al. [73] found that the mortality of *S. oryzae* was significantly higher when silica gel was combined with the EO of *Juniperus oxycedrus* L. ssp. *oxycedrus* (Pinales: Cupressaceae) compared to the silica gel alone. In this scenario, additional experimental efforts are necessary to shed light on the impact of *H. voyronii* EO-based NE as a multi-species killing agent.

## Figures and Tables

**Table 1:** Evaluation of the insecticidal activity of a 6% (*w*/*w*) *Hazomalania voyronii* essential oil nanoemulsion: MANOVA parameters about the main effects and associated interactions leading to the observed mortality rates on *Tribolium castaneum, Tribolium confusum*, and *Tenebrio molitor* adults and larvae, between and within exposure intervals (error *df* = 96).

Between Exposure Intervals	*df*	*F*	*p*
Intercept	1	892.5	<0.01
Nanoemulsion concentration	1	8.4	<0.01
Insect species-stage	5	67.0	<0.01
Nanoemulsion concentration x insect species-stage	5	0.3	0.89
Within exposure intervals	*df*	*F*	*P*
Exposure	9	85.0	<0.01
Exposure x nanoemulsion concentration	9	0.9	0.51
Exposure x insect species-stage	45	6.1	<0.01
Exposure x nanoemulsion concentration x insect species-stage	45	1.1	0.40

**Table 2:** Mean (%) mortality ± SE of *Tribolium castaneum* adults and larvae after 4 h, 8 h, 16 h, 1 day, 2 days, 3 days, 4 days, 5 days, 6 days, and 7 days of exposure to wheat treated with a 6% (*w*/*w*) *Hazomalania voyronii* essential oil nanoemulsion at two concentrations. Within each column, asterisks indicate significant differences, and in all cases *df* = 16; two-tailed *t*-test at *p* = 0.05. Within each row, means followed by the same uppercase letter are not significantly different, *df* = 9, 89; Tukey’s HSD test at *p* = 0.05. Where no letters or no asterisks exist, no significant differences were recorded. Where dashes exist, no statistical analysis was performed.

Exposure	4 h	8 h	16 h	1 Day	2 Days	3 Days	4 Days	5 Days	6 Days	7 Days	*F*	*p*
	Adults											
500 ppm	0.0 ± 0.0 ^Β^	0.0 ± 0.0 ^Β^	0.0 ± 0.0 ^Β^	0.0 ± 0.0 ^Β^	2.2 ± 1.5 ^AΒ^	3.3 ± 2.4 ^AΒ^	3.3 ± 2.4 ^AΒ^	6.7 ± 2.9 ^AΒ^	9.1 ± 4.0 ^AΒ^	12.5 ± 4.7 ^A^	3.1	<0.01
1000 ppm	0.0 ± 0.0 ^B^	0.0 ± 0.0 ^B^	0.0 ± 0.0 ^B^	0.0 ± 0.0 ^B^	2.2 ± 1.5 ^AB^	7.8 ± 3.2 ^AB^	11.4 ± 4.0 ^AB^	13.2 ± 3.8 ^A^	16.5 ± 4.8 ^A^	18.7 ± 5.4 ^A^	6.1	<0.01
*t*	-	-	-	-	0	−1.0	−1.6	−1.1	−1.0	−0.6		
*p*	-	-	-	-	1.00	0.31	0.13	0.29	0.32	0.53		
	Larvae											
500 ppm	2.2 ± 1.5 ^D^	8.9 ± 2.6 ^CD^	10.0 ± 2.9 ^C^	14.4 ± 2.9 ^BC^	30.1 ± 4.6 ^AB^	41.6 ± 5.2 ^AB^	54.4 ± 5.0 ^A^	58.2 ± 5.7 ^A^	60.7 ± 6.8 ^A^	84.1 ± 4.1 ^A^	23.3	<0.01
1000 ppm	4.4 ± 1.8 ^C^	10.0 ± 2.4 ^C^	12.6 ± 3.3 ^BC^	15.1 ± 4.2 ^BC^	30.3 ± 4.9 ^AB^	42.7 ± 5.4 ^A^	54.9 ± 4.5 ^A^	70.1 ± 3.5 ^A^	87.1 ± 3.6 ^A^*	97.4 ± 1.7 ^A^*	19.7	<0.01
*t*	−1.0	−0.5	−0.5	0.4	<0.1	−0.2	−0.1	−1.9	−3.3	−2.9		
*p*	0.35	0.66	0.61	0.72	1.00	0.87	0.89	0.08	<0.01	0.01		

**Table 3:** Mean (%) mortality ± SE of *Tribolium confusum* adults and larvae after 4 h, 8 h, 16 h, 1 day, 2 days, 3 days, 4 days, 5 days, 6 days, and 7 days of exposure to wheat treated with a 6% (*w*/*w*) *Hazomalania voyronii* essential oil nanoemulsion at two concentrations. Within each column, asterisks indicate significant differences, in all cases *df* = 16; two-tailed *t*-test at *p* = 0.05. Within each row, means followed by the same uppercase letter are not significantly different, *df* = 9, 89; Tukey’s HSD test at *p* = 0.05. Where no letters or no asterisks exist, no significant differences were recorded. Where dashes exist, no statistical analysis was performed.

Exposure	4 h	8 h	16 h	1 Day	2 Days	3 Days	4 Days	5 Days	6 Days	7 Days	*F*	*p*
	Adults											
500 ppm	0.0 ± 0.0 ^C^	0.0 ± 0.0 ^C^	0.0 ± 0.0 ^C^	0.0 ± 0.0 ^C^	0.0 ± 0.0 ^C^	0.0 ± 0.0 ^C^	3.3 ± 1.7 ^BC^	7.9 ± 2.8 ^AB^	10.3 ± 2.4 ^A^	10.3 ± 2.4 ^A^	11.3	<0.01
1000 ppm	0.0 ± 0.0 ^B^	0.0 ± 0.0 ^B^	1.1 ± 1.1 ^B^	2.2 ± 2.2 ^B^	3.3 ± 2.4 ^AB^	4.4 ± 2.4 ^AB^	5.6 ± 2.4 ^AB^	9.3 ± 2.0 ^A^	10.8 ± 2.5 ^A^	13.0 ± 3.3 ^A^	5.9	<0.01
*t*	-	-	−1	−1	−1.5	−2.0	−0.6	−0.8	−0.1	−0.3		
*p*	-	-	0.33	0.33	0.15	0.07	0.58	0.44	0.95	0.8		
	Larvae											
500 ppm	0.0 ± 0.0 ^D^	0.0 ± 0.0 ^D^	1.1 ± 1.1 ^D^	4.7 ± 2.6 ^CD^	6.9 ± 3.0 ^CD^	13.0 ± 4.3 ^BC^	20.7 ± 3.0 ^AB^	26.4 ± 1.7 ^A^	41.9 ± 2.3 ^A^	59.3 ± 2.9 ^A^	31.7	<0.01
1000 ppm	0.0 ± 0.0 ^F^	1.1 ± 1.1 ^F^	4.4 ± 2.4 ^EF^	13.3 ± 4.7 ^DE^	21.1 ± 6.1 ^CD^*	28.6 ± 6.2 ^BCD^	36.0 ± 6.2 ^ABC^	45.0 ± 6.7 ^ABC^*	67.7 ± 2.3 ^AB^*	92.1 ± 2.0 ^A^*	29.2	<0.01
*t*	-	−1	−1.2	−1.6	−2.3	−1.7	−1.9	−2.2	−7.6	−8.7		
*p*	-	0.33	0.26	0.14	0.03	0.12	0.07	0.05	<0.01	<0.01		

**Table 4:** Mean (%) mortality ± SE of *Tenebrio molitor* adults and larvae after 4 h, 8 h, 16 h, 1 day, 2 days, 3 days, 4 days, 5 days, 6 days, and 7 days of exposure to wheat treated with a 6% (*w*/*w*) *Hazomalania voyronii* essential oil nanoemulsion at two concentrations. Within each column, asterisks indicate significant differences, in all cases *df* = 16; two-tailed *t*-test at *P* = 0.05. Within each row, means followed by the same uppercase letter are not significantly different, *df* = 9, 89; Tukey’s HSD test at *p* = 0.05. Where no letters or no asterisks exist, no significant differences were recorded. Where dashes exist, no statistical analysis was performed.

Exposure	4 h	8 h	16 h	1 Day	2 Days	3 Days	4 Days	5 Days	6 Days	7 Days	*F*	*p*
	Adults											
500 ppm	0.0 ± 0.0 ^E^	0.0 ± 0.0 ^E^	6.8 ± 2.4 ^D^	15.8 ± 4.4 ^CD^	25.9 ± 4.7 ^BC^	43.1 ± 9.3 ^AB^	55.3 ± 6.1 ^AB^	70.8 ± 6.0 ^A^	80.1 ± 4.9 ^A^	94.8 ± 2.8 ^A^	59.6	<0.01
1000 ppm	0.0 ± 0.0 ^E^	0.0 ± 0.0 ^E^	9.0 ± 2.0 ^D^	21.7 ± 5.2 ^CD^	41.3 ± 8.0 ^BC^	63.3 ± 9.1 ^AB^	72.5 ± 8.2 ^AB^	84.3 ± 5.9 ^AB^	93.5 ± 3.8 ^AB^*	100.0 ± 0.0 ^A^	49.1	<0.01
*t*	-	-	−0.9	−0.4	−0.4	−1.4	−1.3	−1.4	−2.1	−1.9		
*p*	-	-	0.38	0.68	0.68	0.17	0.21	0.18	0.05	0.08		
	Larvae											
500 ppm	0.0 ± 0.0^B^	0.0 ± 0.0 ^B^	0.0 ± 0.0 ^B^	0.0 ± 0.0 ^B^	0.0 ± 0.0 ^B^	1.1 ± 1.1 ^AB^	2.2 ± 1.5 ^AB^	3.3 ± 1.7 ^AB^	5.6 ± 2.4 ^A^	5.8 ± 2.5 ^A^	3.0	<0.01
1000 ppm	0.0 ± 0.0 ^C^	0.0 ± 0.0 ^C^	0.0 ± 0.0 ^C^	0.0 ± 0.0 ^C^	4.4 ± 2.4 ^B^	4.4 ± 2.4 ^B^	5.6 ± 2.4 ^AB^	5.6 ± 2.4 ^AB^	5.6 ± 2.4 ^AB^	10.3 ± 3.8 ^A^	3.0	<0.01
*t*	-	-	-	-	−2.0	−1.2	−1.1	−0.6	0	−0.7		
*p*	-	-	-	-	0.07	0.26	0.31	0.58	1.00	0.52		

## Data Availability

All data are within this manuscript.

## Sample Availability

Not available.

## References

[1] Rees D. (2004). Insects of Stored Products.

[2] Hagstrum D.W., Subramanyam B. (2009). Stored-Product Insect Resource.

[3] Robinson W.H. (2005). Urban Insects and Arachnids.

[4] Yezerki A., Gilmor T.P., Stevens L. (2004). Genetic analysis of benzoquinone production in *Tribolium confusum*. J. Chem. Ecol..

[5] Kavallieratos N.G., Andrić G., Golić M.P., Nika E.P., Skourti A., Kljajić P., Papanikolaou N.E. (2020). Biological features and population growth of two Southeastern European *Tribolium confusum* Jacquelin du Val (Coleoptera: Tenebrionidae) strains. Insects.

[6] Krinsky W.L., Mullen G.R., Durden L.A. (2019). Beetles (Coleoptera). Medical and Veterinary Entomology.

[7] Skourti A., Kavallieratos N.G., Papanikolaou N.E. (2019). Laboratory evaluation of development and survival of *Tribolium castaneum* (Herbst) (Coleoptera: Tenebrionidae) under constant temperatures. J. Stored Prod. Res..

[8] Skourti A., Kavallieratos N.G., Papanikolaou N.E. (2020). Suitability of semolina, cracked wheat and cracked maize as feeding commodities for *Tribolium castaneum* (Herbst; Coleoptera: Tenebrionidae). Insects.

[9] Hill D.S. (2003). Pests of Storage Foodstuffs and their Control.

[10] DeFoliart G.R., Resh V.H., Cardé R.T. (2009). Food, Insects as. Encyclopedia of Insects.

[11] Vigneron A., Jehan C., Rigaud T., Moret Y. (2019). Immune defenses of a beneficial pest: The mealworm beetle *Tenebrio molitor*. Front. Physiol..

[12] Arthur F.H. (1996). Grain Protectants: Current status and prospects for the future. J. Stored Prod. Res..

[13] Khaliq A., Ullah M.I., Afzal M., Ali A., Sajjad A., Ahmad A., Khalid S. (2020). Management of *Tribolium castaneum* using synergism between conventional fumigant and plant essential oils. Int. J. Trop. Insect Sc..

[14] Cui K., Zhang L., He L., Zhang Z., Zhang T., Mu W., Lin J., Liu F. (2021). Toxicological effects of the fungal volatile compound 1-octen-3-ol against the red flour beetle, *Tribolium castaneum* (Herbst). Ecotox. Environ. Safe..

[15] Giunti G., Palermo D., Laudani F., Algeri G.M., Campolo O., Palmeri V. (2019). Repellence and acute toxicity of a nano-emulsion of sweet orange essential oil toward two major stored grain insect pests. Ind. Crops Prod..

[16] Giunti G., Campolo O., Laudani F., Zappalà L., Palmeri V. (2021). Bioactivity of essential oil-based nano-biopesticides toward *Rhyzopertha dominica* (Coleoptera: Bostrichidae). Ind. Crops Prod..

[17] Petrović M., Popović A., Kojić D., Šućur J., Bursić V., Aćimović M., Malenčić Đ., Stojanović T., Vuković G. (2019). Assessment of toxicity and biochemical response of *Tenebrio molitor* and *Tribolium confusum* exposed to *Carum carvi* essential oil. Entomol. Gen..

[18] Kavallieratos N.G., Boukouvala M.C., Ntalli N., Kontodimas D.C., Cappellacci L., Petrelli R., Ricciutelli M., Benelli G., Maggi F. (2020). Efficacy of the furanosesquiterpene isofuranodiene against the stored-product insects *Prostephanus truncatus* (Coleoptera: Bostrychidae) and *Trogoderma granarium* (Coleoptera: Dermestidae). J. Stored Prod. Res..

[19] Hashem A.S., Awadalla S.S., Zayed G.M., Maggi F., Benelli G. (2018). *Pimpinella anisum* essential oil nanoemulsions against *Tribolium castaneum*—insecticidal activity and mode of action. Environ. Sci. Pollut. Res..

[20] Kavallieratos N.G., Boukouvala M.C., Ntalli N., Skourti A., Karagianni E.S., Nika E.P., Kontodimas D.C., Cappellacci L., Petrelli R., Cianfaglione K. (2020). Effectiveness of eight essential oils against two key stored-product beetles, *Prostephanus truncatus* (Horn) and *Trogoderma granarium* Everts. Food Chem. Toxicol..

[21] Athanassiou C.G., Kavallieratos N.G., Benelli G., Losic D., Rani P.U., Desneux N. (2018). Nanoparticles for pest control: Current status and future perspectives. J. Pest Sci..

[22] Benelli G. (2020). On a magical mystery tour of green insecticide research: Current issues and challenges. Molecules.

[23] Pavoni L., Pavela R., Cespi M., Bonacucina G., Maggi F., Zeni V., Canale A., Lucchi A., Bruschi F., Benelli G. (2019). Green micro- and nanoemulsions for managing parasites, vectors and pests. Nanomaterials.

[24] Turek C., Stintzing F.C. (2013). Stability of essential oils: A review. Compr. Rev. Food Sci. Food Saf..

[25] Pavela R., Pavoni L., Bonacucina G., Cespi M., Kavallieratos N.G., Cappellacci L., Petrelli R., Maggi F., Benelli G. (2019). Rationale for developing novel mosquito larvicides based on isofuranodiene microemulsions. J. Pest Sci..

[26] Pavela R., Benelli G., Pavoni L., Bonacucina G., Cespi M., Cianfaglione K., Bajalan I., Morshedloo M.R., Lupidi G., Romano D. (2019). Microemulsions for delivery of Apiaceae essential oils—Towards highly effective and eco-friendly mosquito larvicides?. Ind. Crops Prod..

[27] Benelli G., Pavoni L., Zeni V., Ricciardi R., Cosci F., Cacopardo G., Gendusa S., Spinozzi E., Petrelli R., Cappellacci L. (2020). Developing a highly stable *Carlina acaulis* essential oil nanoemulsion for managing *Lobesia botrana*. Nanomaterials.

[28] Heydari M., Amirjani A., Bagheri M., Sharifian I., Sabahi Q. (2020). Eco-friendly pesticide based on peppermint oil nanoemulsion: Preparation, physicochemical properties, and its aphicidal activity against cotton aphid. Environ. Sci. Pollut. Res..

[29] Rossi P., Cappelli A., Marinelli O., Valzano M., Pavoni L., Bonacucina G., Petrelli R., Pompei P., Mazzara E., Ricci I. (2020). Mosquitocidal and anti-inflammatory properties of the essential oils obtained from monoecious, male, and female inflorescences of hemp (*Cannabis sativa* L.) and their encapsulation in nanoemulsions. Molecules.

[30] Ghosh V., Mukherjee A., Chandrasekaran N. (2013). Formulation and characterization of plant essential oil based nanoemulsion: Evaluation of its larvicidal activity against *Aedes aegypti*. Asian J. Chem..

[31] Duarte J.L., Amado J.R.R., Oliveira A.E.M.F.M., Cruz R.A.S., Ferreira A.M., Souto R.N.P., Falcão D.Q., Carvalho J.C.T., Fernandes C.P. (2015). Evaluation of larvicidal activity of a nanoemulsion of *Rosmarinus officinalis* essential oil. Rev. Bras. Farmacogn..

[32] Pavela R., Pavoni L., Bonacucina G., Cespi M., Cappellacci L., Petrelli R., Spinozzi E., Aguzzi C., Zeppa L., Ubaldi M. (2021). Encapsulation of *Carlina acaulis* essential oil and carlina oxide to develop long-lasting mosquito larvicides: Microemulsions versus nanoemulsions. J. Pest Sci..

[33] Lima L.A., Ferreira-Sá P.S., Garcia M.D., Pereira V.L.P., Carvalho J.C.T., Rocha L., Fernandes C.P., Souto R.N.P., Araújo R.S., Botas G. (2021). Nano-emulsions of the essential oil of *Baccharis reticularia* and its constituents as eco-friendly repellents against *Tribolium castaneum*. Ind. Crops Prod..

[34] Benelli G., Pavela R., Rakotosaona R., Nzekoue F.K., Canale A., Nicoletti M., Maggi F. (2020). Insecticidal and mosquito repellent efficacy of the essential oils from stem bark and wood of *Hazomalania voyronii*. J. Ethnopharmacol..

[35] Hobbs C.A., Taylor S.V., Beevers C., Lloyd M., Bowen R., Lillford L., Maronpot R., Hayashi S. (2016). Genotoxicity assessment of the flavouring agent, perillaldehyde. Food Chem. Toxicol..

[36] Oser B.L., Ford R.A. (1978). Recent progress in the consideration of flavoring ingredients under the Food Additives Amendment. 11. GRAS substances. Food Technol..

[37] Kavallieratos N.G., Michail E.J., Boukouvala M.C., Nika E.P., Skourti A. (2019). Efficacy of pirimiphos-methyl, deltamethrin, spinosad and silicoSec against adults and larvae of *Tenebrio molitor* L. on wheat, barley and maize. J. Stored Prod. Res..

[38] Kavallieratos N.G., Papanikolaou N.E., Kazani A.N., Boukouvala M.C., Malesios C. (2020). Using multilevel models to explore the impact of abiotic and biotic conditions on the efficacy of pirimiphos-methyl against *Tenebrio molitor* L. Environ. Sci. Pollut. Res..

[39] Kathirvelu C., Maline A.S., Raja B.A.G., Kavitha T. (2020). Effect of fumigation on *Rhyzopertha dominica* F. and *Tribolium castaneum* H. in stored products using essential oils. Ann. Agri Bio Res..

[40] Lee H.K., Jeong G., Kim H.K., Kim B.S., Yang J.O., Koo H.N., Kim G.H. (2020). Fumigation activity against phosphine-resistant *Tribolium castaneum* (Coleoptera: Tenebrionidae) using carbonyl sulfide. Insects.

[41] Arthur F.H., Carvalho M.O., Fields P.G., Adler C.S., Arthur F.H., Athanassiou C.G., Campbell J.F., Fleurat-Lessard F., Flinn P.W., Hodges R.J., Isikber A.A. (2010). Residual efficacy of aerosols to control *Tribolium castaneum* and *Tribolium confusum.*. Proceedings of the 10th International Working Conference on Stored Product Protection.

[42] Deb M., Kumar D. (2020). Bioactivity and efficacy of essential oils extracted from *Artemisia annua* against *Tribolium castaneum* (Herbst. 1797) (Coleoptera: Tenebrionidae): An eco-friendly approach. Ecotox. Environ. Saf..

[43] Mujeeb K.A., Shakoori A.R. (2012). Effect of an organophosphate, pirimiphos-methyl, on esterases of different developmental stages of stored grain pest red flour beetle, *Tribolium castaneum* (Herbst.)—spectrophotometric analysis. Pak. J. Zool..

[44] Vayias B.J., Athanassiou C.G. (2004). Factors affecting the insecticidal efficacy of the diatomaceous earth formulation silicoSec against adults and larvae of the confused flour beetle, *Tribolium confusum* DuVal (Coleoptera: Tenebrionidae). Crop Prot..

[45] Athanassiou C.G., Kavallieratos N.G. (2014). Evaluation of spinetoram and spinosad for control of *Prostephanus truncatus*, *Rhyzopertha dominica*, *Sitophilus oryzae*, and *Tribolium confusum* on stored grains under laboratory tests. J. Pest Sci..

[46] Boukouvala M.C., Kavallieratos N.G., Athanassiou C.G., Hadjiarapoglou L.P. (2016). Biological activity of two new pyrrole derivatives against stored-product species: Influence of temperature and relative humidity. Bull. Entomol. Res..

[47] Boukouvala M.C., Kavallieratos N.G., Athanassiou C.G., Hadjiarapoglou L.P. (2016). Insecticidal effect of two novel pyrrole derivatives against two major stored product insect species. Crop Prot..

[48] Boukouvala M.C., Kavallieratos N.G., Athanassiou C.G., Benelli G., Hadjiarapoglou L.P. (2019). Insecticidal efficacy of six new pyrrole derivatives against four stored-product pests. Environ. Sci. Pollut. Res..

[49] Jankowska M., Rogalska J., Wyszkowska J., Stankiewicz M. (2018). Molecular targets for components of essential oils in the insect nervous system—A review. Molecules.

[50] Park C.G., Jang M., Yoon K.A., Kim J. (2016). Insecticidal and acetylcholinesterase inhibitory activities of Lamiaceae plant essential oils and their major components against *Drosophila suzukii* (Diptera: Drosophilidae). Ind. Crops Prod..

[51] You C.X., Wang Y., Zhang W.J., Yang K., Wu Y., Geng Z.F., Chen H.P., Jiang H.Y., Du S.S., Deng Z.W. (2014). Chemical constituents and biological activities of the purple *Perilla* essential oil against *Lasioderma serricorne*. Ind. Crops Prod..

[52] Traboulsi A.F., El-Haj S., Tueni M., Taoubi K., Nader N.A., Mrad A. (2005). Repellency and toxicity of aromatic plant extracts against the mosquito *Culex pipiens molestus* (Diptera: Culicidae). Pest Manag. Sci..

[53] Kordali S., Aslan I., Calmasur O., Cakir A. (2006). Toxicity of essential oils isolated from three *Artemisia species* and some of their major components to granary weevil *Sitophilus granarius* (L.) (Coleoptera: Curculinonidae). Ind. Crops Prod..

[54] Kumar P., Mishra S., Malik A., Satya S. (2013). Housefly (*Musca domestica* L.) control potential of *Cymbopogon citratus* Stapf. (Poales: Poaceae) essential oil and monoterpenes (citral and 1, 8-cineole). Parasitol. Res..

[55] Maggi F., Benelli G. (2018). Essential oils from aromatic and medicinal plants as effective weapons against mosquito vectors of public health importance. Mosquito-borne Diseases.

[56] Nansen C., Subramanyam B., Roesli R. (2004). Characterizing spatial distribution of trap captures of beetles in retail pet stores using SADIE^®^ software. J. Stored Prod. Res..

[57] Athanassiou C.G., Kavallieratos N.G., Sciarretta A., Palyvos N.E., Trematerra P. (2011). Spatial associations of insects and mites in stored wheat. J. Econ. Entomol..

[58] Arthur F.H., Campbell J.F., Toews M.D. (2014). Distribution, abundance, and seasonal patterns of stored product beetles in a commercial food storage facility. J. Stored Prod. Res..

[59] Kavallieratos N.G., Athanassiou C.G., Guedes R.N.C., Drempela J.D., Boukouvala M.C. (2017). Invader competition with local competitors: Displacement or coexistence among the invasive khapra beetle, *Trogoderma granarium* Everts (Coleoptera: Dermestidae), and two other major stored-grain beetles?. Front. Plant Sci..

[60] Nika E.P., Kavallieratos N.G., Papanikolaou N.E. (2021). Linear and non-linear models to explain influence of temperature on life history traits of *Oryzaephilus surinamensis* (L.). Entomol. Gen..

[61] De Vosjoli P. (2007). The Lizard Keeper’s Handbook.

[62] Kavallieratos N.G., Athanassiou G.G., Boukouvala M.C. (2013). Insecticidal effect of chlorantraniliprole against major stored-product insect pests in different grain commodities under laboratory tests. Pest Manag. Sci..

[63] Boukouvala M.C., Romano D., Kavallieratos N.G., Athanassiou C.G., Stefanini C., Canale A., Benelli G. (2019). Asymmetric courtship boosts male mating success in the red flour beetle, *Tribolium castaneum* (Herbst) (Coleoptera: Tenebrionidae). J. Stored Prod. Res..

[64] Cappellani M.R., Perinelli D.R., Pescosolido L., Schoubben A., Cespi M., Cossi R., Blasi P. (2018). Injectable nanoemulsions prepared by high pressure homogenization: Processing, sterilization, and size evolution. Appl. Nanosci..

[65] Abbott W.S. (1925). A Method of Computing the Effectiveness of an Insecticide. J. Econ. Entomol..

[66] Zar J.H. (2010). Biostatistical analysis.

[67] Scheff D.S., Arthur F.H. (2018). Fecundity of *Tribolium castaneum* and *Tribolium confusum* adults after exposure to deltamethrin packaging. J. Pest Sci..

[68] Sall J., Lehman A., Creighton L. (2001). JMP Start Statistics. A Guide to Statistics and Data Analysis Using JMP and JMP IN Software.

[69] SAS Institute Inc (2018). Using JMP 14.

[70] Sokal R.R., Rohlf F.J. (1995). Biometry.

[71] Snedecor G.W., Cochran W.G. (1980). Statistical Methods.

[72] Andrić G., Marković M.M., Adamović M., Daković A., Golić M.P., Kljajić P.J. (2012). Insecticidal potential of natural zeolite and diatomaceous earth formulations against rice weevil (Coleoptera: Curculionidae) and red flour beetle (Coleoptera: Tenebrionidae). J. Econ. Entomol..

[73] Athanassiou C.G., Kavallieratos N.G., Evergetis E., Katsoula A.M., Haroutounian S. (2013). Insecticidal efficacy of silica gel with *Juniperus oxycedrus* ssp. oxycedrus (Pinales: Cupressaceae) essential oil against Sitophilus oryzae (Coleoptera: Curculionidae) and Tribolium confusum (Coleoptera: Tenebrionidae). J. Econ. Entomol..

